# Comparison of osteogenic differentiation capacity in mesenchymal stem cells derived from human amniotic membrane (AM), umbilical cord (UC), chorionic membrane (CM), and decidua (DC)

**DOI:** 10.1186/s13578-019-0281-3

**Published:** 2019-02-11

**Authors:** Chongyang Shen, Chuan Yang, Shijun Xu, Hai Zhao

**Affiliations:** 1 Basic Medicine School, Chengdu University of Traditional Chinese Medicine, Chengdu, People’s Republic of China; 2 Key Laboratory of Obstetric and Gynecologic and Pediatric Diseases and Birth Defects of the Ministry of Education, West China Second University Hospital, Sichuan University, Chengdu, People’s Republic of China; 3 College Pharmacy, Chengdu University of Traditional Chinese Medicine, Chengdu, People’s Republic of China; 4 Department of Neurosurgery, West China Hospital, Sichuan University, Chengdu, People’s Republic of China

**Keywords:** Mesenchymal stem cells, Osteogenic differentiation, Fibronectin, Kinetics, Laminin

## Abstract

**Background:**

Mesenchymal stem cells (MSCs) have been extensively explored as a promising therapeutic agent in the field of bone tissue engineering due to their osteogenic differentiation ability. In this study, the osteogenic differential ability and the effect of fibronectin and laminin on the osteogenic differentiation in four types of MSCs derived from placental tissue are compared to determine the ideal source for bone reconstruction tissue engineering.

**Results:**

The present study examines the osteogenic differentiation levels of four types of MSCs using alizarin red staining and quantifies the calcium levels and alkaline phosphatase (ALP) activity. In addition, this study examines the osteoblast differentiation protein markers osterix, collagen I, osteopontin, and osteocalcin using a Western blot assay. qPCR and EdU labeling assays were employed to identify the kinetics of osteogenic differentiation. Calcium deposit levels, ALP activity, and osteopontin and osteocalcin concentrations were determined to confirm the role of Extracellular matrix (ECM) components role on the osteogenic differentiation of MSCs. The data demonstrated that MSCs isolated from different layers of placenta had different potentials to differentiate into osteogenic cells. Importantly, AM-MSCs and UC-MSCs differentiated into the osteoblast stage more efficiently and quickly than CM-MSCs and DC-MSCs, which was associated with a decrease in their proliferation ability. Among the different types of MSCs, AM-MSCs and UC-MSCs had a higher osteogenic differentiation potential induced by fibronectin due to enhanced phosphorylation during the Akt and ERK pathways.

**Conclusions:**

Taken together, these results indicate that AM-MSCs and UC-MSCs possess a higher osteogenic potential, and fibronectin can robustly enhance the osteogenic potential of the Akt and ERK pathways.

**Electronic supplementary material:**

The online version of this article (10.1186/s13578-019-0281-3) contains supplementary material, which is available to authorized users.

## Background

Bones have a healing capacity due to their ability to build bone bridges in gaps in response to a minor injury, however, severely damaged bone or critically-sized defects in bone lead to a failure in the gap healing process, and in these cases, bone graft surgical procedures are required [1]. Bone grafts are the second most transplanted tissue in the world behind blood. Despite recent advances in medical care, bone implants do have limitations, such as bad biocompatibility and mechanical integrity [2]. Stem cell-based bone tissue engineering, using a combination of stem cells, biomaterials, and bioactive macro-molecules, is the new frontier for the reconstruction of bone defects [3]. These approaches typically count on the use of stem cells, where stem cells are modified to differentiate to osteoblasts for bone replacement or fill implants.

For years, MSCs have been considered to be multipotent stem cells that are capable of self-renewal and differentiation into a variety of cell types, including osteoblasts, chondroblasts, adipocytes [4], cardiomyocytes [5], and pancreatic β cells [6]. MSCs currently can be extracted from different adult and fetal tissues, such as adipose tissue [7], bone marrow [8], dental pulp [9], and placenta tissue [10]. In the early stages of development, adipose tissue-derived MSCs (ADSCs) and bone marrow MSCs (BM-MSCs) have been the paramount agents of clinical cytotherapy. However, two barriers exist to the successful application of these MSCs. First, the isolation of adipose tissue and bone marrow are invasive procedures. Second, ADSCs and BM-MSCs exhibit a significant proliferative rate decline, short life span, and reduced differentiation capacity with increasing age and in several disease phenotypes [11, 12].

Placental tissue can be easily obtained, and usually it is considered as medical waste. It is becoming increasingly appreciated that placenta is the most important source of MSCs. Placental tissue is composed of a variety of tissues, including umbilical cord, amnion, chorion and diaphragm tissues. Recently, several studies have isolated and identified MSCs from placental tissues, including the amniotic membrane (AM) [13], the umbilical cord (UC) [14], the chorionic membrane (CM) [15], and the decidua (DC) [16]. These cells, as stem cells, share the common characteristics of self-renewal, rapid proliferation, and multipotency. Other recent studies, however, have shown different morphologies and multifaceted roles in cytotherapy. Amniotic membrane mesenchymal stem cells (AM-MSCs) have been shown to have a high potential to differentiate into cardiomyocytes and display immunologic tolerance in vivo [17]. Umbilical cord mesenchymal stem cells (UC-MSCs) have been shown to have high proliferative ability and the capacity to differentiate into osteogenic phenotypes [18]. Upon exposure to a neuronal differentiation medium, chorionic membrane mesenchymal stem cells (CM-MSCs) changed their cell morphology and differentiated into neuron like cells [19]. Decidua mesenchymal stem cells (DC-MSCs) have been shown to have an immunomodulatory effect in vivo [20].

MSCs with osteogenic potential have been extensively studied since 2001 [21, 22]. Introducing ECM components to stem cells-based tissue engineering field not only provide cellular structural support, but also provide the biochemical cues to facilitate and orchestrate cell physiology and phenotype [23]. Placental-derived MSCs (PD-MSCs) are attractive candidates for cell-based bone tissue engineering, but the osteogenic differentiation dynamics and the diversity of PD-MSCs phenotypes during the interaction of extracellular matrix proteins, such as fibronectin (FN) and laminin (LAM), in controlling cell differentiation are worthy of substantial additional investigation.

Given the complexity of MSCs differentiation processes into osteogenic lineages, the current strategies of stem cell-based regeneration for the prevention and/or treatment of human diseases such as bone nonunion, bone defects and osteoporosis, require careful evaluation of the osteogenic differentiation potential between AM-MSCs, UC-MSCs, CM-MSCs, and DC-MSCs.

## Results

### Osteogenic potential of AM-MSCs, UC-MSCs, CM-MSCs, and DC-MSCs

**Fig. 1:**
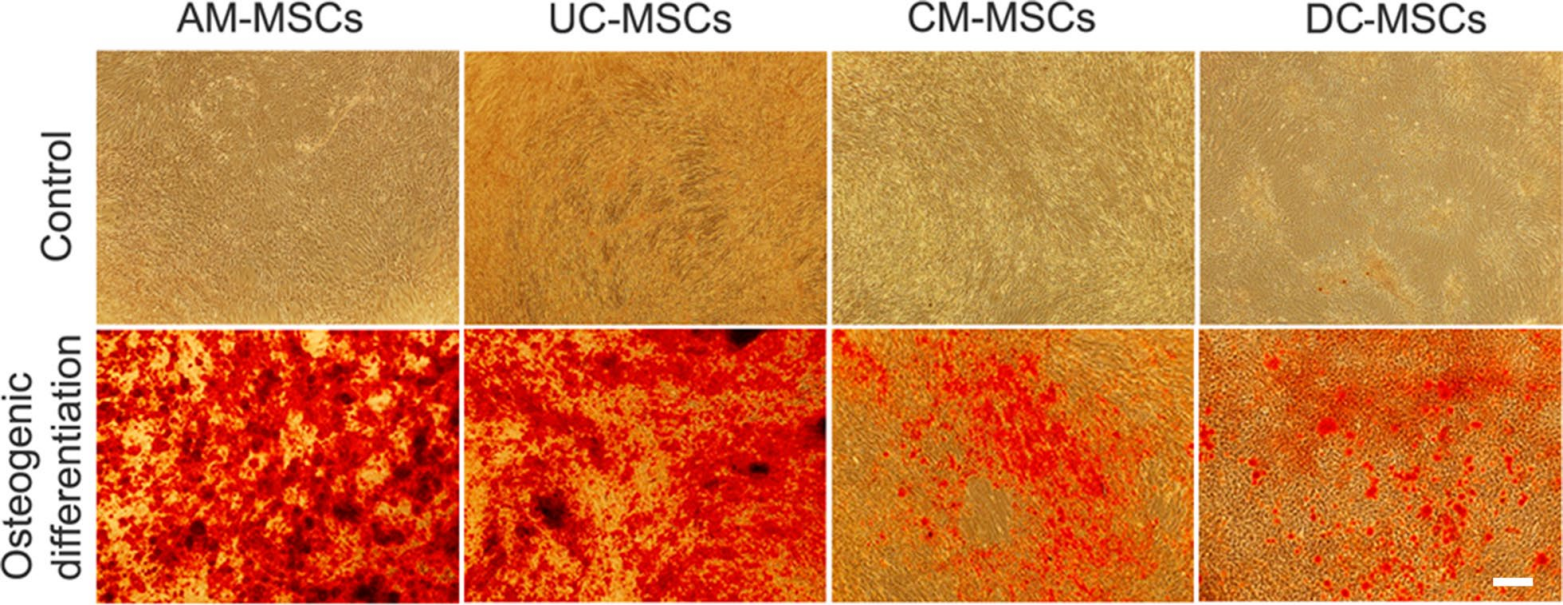
Cell morphology and alizarin red staining of AM-MSCs, UC-MSCs, CM-MSCs, and DC-MSCs after 21 days of osteogenic differentiation. AM-MSCs, UC-MSCs, CM-MSCs, and DC-MSCs cultures with α-MEM for 21 days. Alizarin red staining for analysis of the calcium deposition amount of the AM-MSCs, UC-MSCs, CM-MSCs, and DC-MSCs after 21 days of osteogenic differentiation. Scale bar = 500 μm, magnification = ×40

**Fig. 2:**
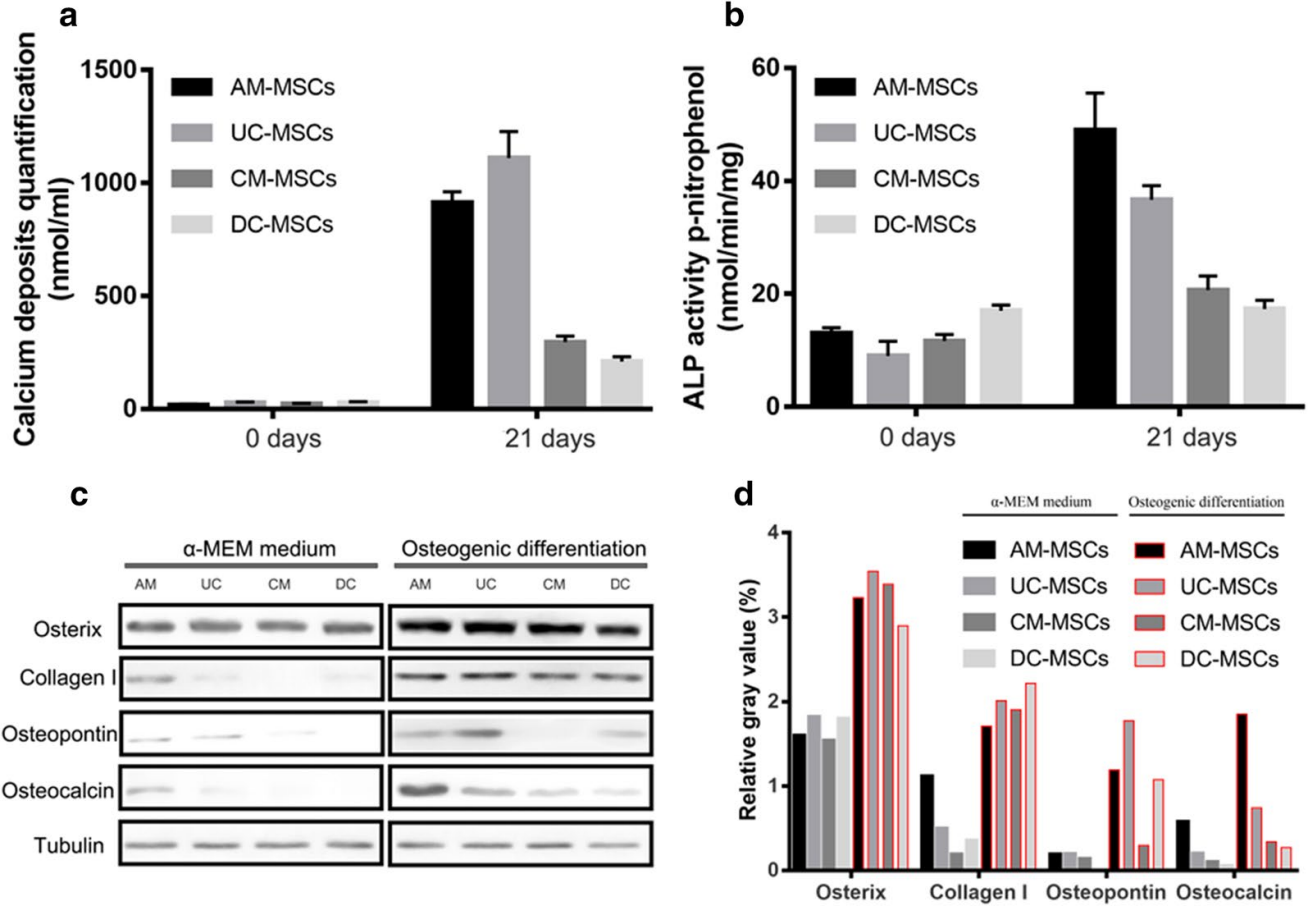
Calcium deposit levels and ALP activity after osteogenic differentiation of AM-MSCs, UC-MSCs, CM-MSCs, and DC-MSCs. **a** Calcium deposit levels and **b** ALP activity were measured spectrophotometrically at days 0 and 21. n = 3/group, Error bars represent the SD, *p < 0.05, **P ≤ 0.01; ***p ≤ 0.001 in different groups compared with the control group by the One-way ANOVA test. **c** Western blot analysis of osteoblast markers (osterix, collagen I, osteopontin, and osteocalcin) proteins in AM-MSCs, UC-MSCs, CM-MSCs, and DC-MSCs with and without 21 days of osteogenic differentiation. **d** The relative gray value of osterix, collagen I, osteopontin, and osteocalcin/tubulin

To determine the osteogenic differentiation levels of AM-MSCs, UC-MSCs, CM-MSCs, and DC-MSCs, dexamethasone and β-glycerophosphate based osteogenic cell induction was performed in which each of the cells was individually cultured in an osteogenic differentiation medium for 21 days. As shown in (Fig. 1), AM-MSCs and UC-MSCs showed more intensive alizarin red staining than the undifferentiated groups. In contrast, much weaker and fewer alizarin red staining was detected in the CM-MSCs and DC-MSCs groups. To quantify the calcium levels of the deposited matrix in these cells, deposited calcium was dissolved in HCl and quantified using a spectrophotometer (Fig. 2a). As expected, calcium levels were markedly enhanced in the AM-MSCs and UC-MSCs compared with the CM-MSCs, and DC-MSCs. The expression of another osteogenic marker, ALP, also dramatically increased after induction in the AM-MSCs and UC-MSCs compared with the control cells (Fig. 2b). ALP activity in the CM-MSCs and DC-MSCs were slightly promoted compared to the undifferentiated cells.

The osteoblast differentiation protein markers osterix, collagen I, osteopontin, and osteocalcin were examined using a Western blot assay (Fig. 2c). Osterix and collagen I was expressed at higher levels in all of the cell types compared with control cells 21 days after differentiation. The protein level of osteopontin was significantly higher in the AM-MSCs, UC-MSCs, and DC-MSCs than in the control cells, with the exception of the CM-MSCs, which showed no difference between the differentiated and undifferentiated status (Fig. 2d). In addition, the osteogenic marker, osteocalcin, was strongly enhanced in the AM-MSCs and UC-MSCs. Differentiated CM-MSCs and DC-MSCs cells only showed slightly higher levels of protein than the control cells (Fig. 2d). Together, these findings indicate that mesenchymal stem cells divided from different placenta tissue exhibited a unique osteoblast differentiation potential, and there was a significant difference between them regarding their osteogenic differentiation related gene expression.

### Kinetics of osteogenic differentiation of AM-MSCs, UC-MSCs, CM-MSCs, and DC-MSCs

**Fig. 3:**
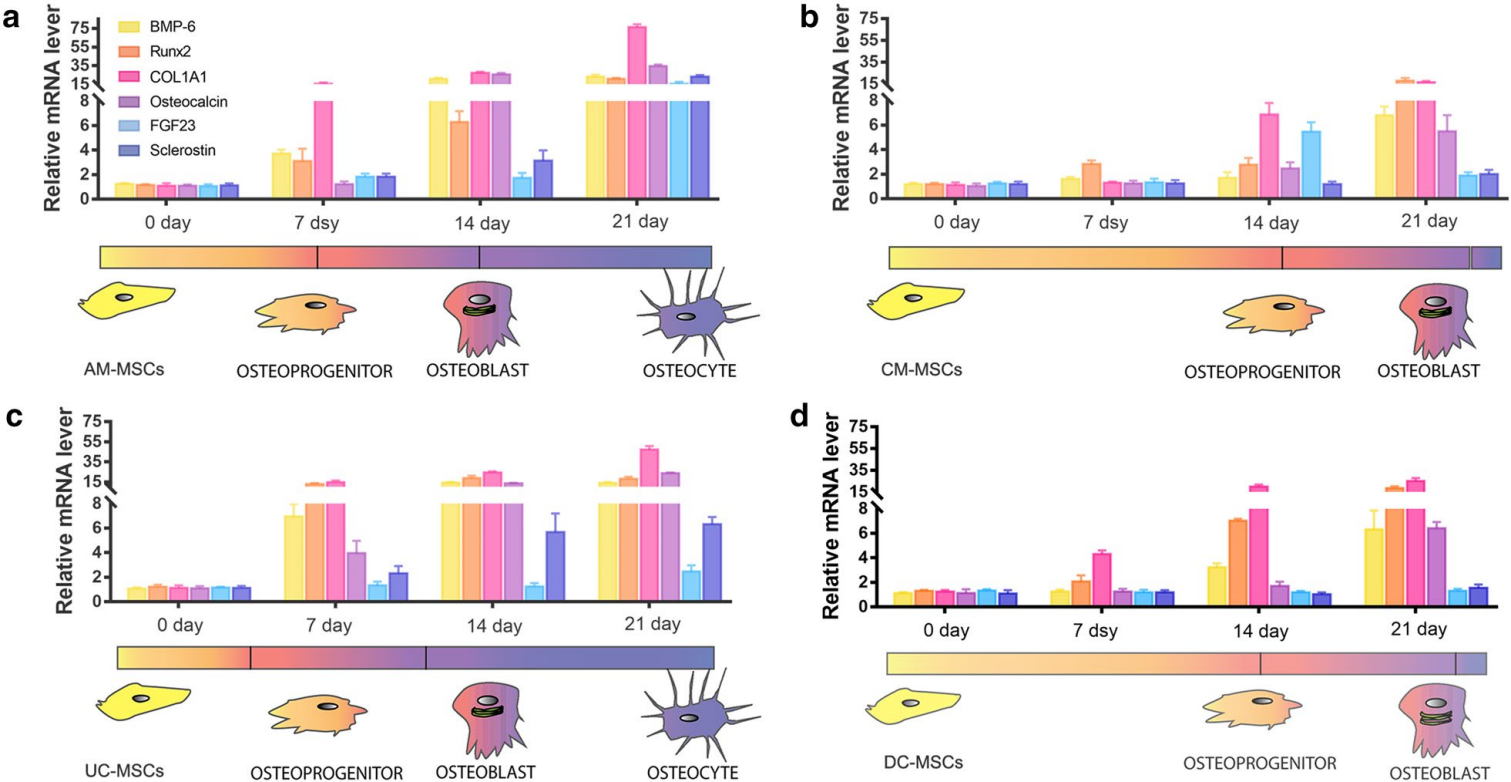
qRT-PCR was performed to analyze the potency and kinetics of osteogenic differentiation in **a** the AM-MSCs, **b** CM -MSCs, **c** UC -MSCs, and **d** DC-MSCs at 0, 7, 14, and 21 days. The y-axis represents the relative mRNA fold change, which was calculated using the 2^−ΔΔCt^ formula with β-actin as internal control. Error bars represent the SD. The colored bar indicates the differentiation processes of PD-MSCs into osteogenic lineages. The yellow bar represents the undifferentiation statue, the red bar represents the osteoprogenitor statue (BMP-6 and Runx2), the purple bar represents the osteoblast statue (COL1A1 and osteocalcin), and the blue bar represents the osteocyte statue (FGF23 and Sclerostin)

Another focus of this study is the kinetics of the expression of osteogenic markers, including osteoprogenitors marker genes (BMP-6, and Runx2), osteoblast markers (COL1A1, and osteocalcin) and osteocyte markers (FGF23, and Sclerostin). As shown in (Fig. 3), at day 7 after osteogenic differentiation, the AM-MSCs and UC-MSCs showed similar BMP-6 and Runx2 mRNA levels, which were clearly higher than that in the CM-MSCs and DC-MSCs, indicating that the development of the AM-MSCs and UC-MSCs began with the commitment into osteoprogenitor cells. With an increase in the differentiation time, the expression of osteoblast marker genes (COL1A1, osteocalcin) in the AM-MSCs and UC-MSCs reached the highest level at day 14, and the cells expressed continuous BMP-6, and Runx2 generation throughout osteogenesis. In contrast, the CM-MSCs and DC-MSCs only partially differentiated into osteoblasts with no significant changes in the expression levels of osteocalcin, but the levels of COL1A1 increased somewhat, and the levels of BMP-6 and Runx2 increased significantly. During the final stages, when osteoblasts transform to fully differentiated osteocytes, elevated expression of FGF23 and sclerostin occurs. The AM-MSCs had the most abundant FGF23 and sclerostin mRNA levels on day 21 after osteogenic differentiation. However, the induction only triggered lower osteocyte differentiation in the UC-MSCs, with sclerostin mRNA expression compared to the AM-MSCs. In addition, FGF23 and sclerostin mRNA showed no significant differences during osteogenic induction in the CM-MSCs and DC-MSCs, but the mRNA expression levels of COL1A1 and osteocalcin on day 21 were significantly higher than that at day 14, which reveals that the stage of osteoblast differentiation from CM-MSCs to DC-MSCs needed more time than in the AM-MSCs and UC-MSCs.

**Fig. 4:**
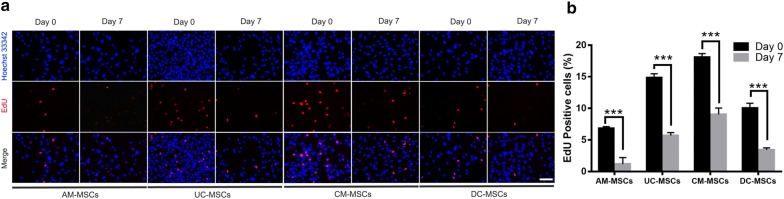
Osteogenic differentiation inhibits cellular proliferation. **a** Cell proliferation was assessed using an EdU labeling assay before and after osteogenic differentiation in the AM-MSCs, UC-MSCs, CM-MSCs, and DC-MSCs. The images display EdU staining (red color) merged with Hoechst 33342 staining (blue color). Scale bar = 100 μm. **b** The graph represents the percentage of EdU-positive cells. Error bars represent the SD, ***p ≤ 0.001 for differences between the two experimental groups were applied using an unpaired two-tailed Student’s t-test

To investigate the changes in the cell’s ability to proliferate during osteogenic differentiation, an EdU labeling assay was assessed (Fig. 4a), which is frequently used as an efficient method to label actively dividing cells. This assessment showed that the percentage of EdU-positive cells in the AM-MSCs and UC-MSCs was markedly decreased when they began to express osteoprogenitors markers genes, such as BMP-6 and Runx2, at day 7 (Fig. 4b). Interestingly, there was also a decline of EdU-positive cells in the CM-MSCs and DC-MSCs in the first 7 days of culture, followed by only a slight increase in the expression of BMP-6 and Runx2. These results indicate that under osteogenic differentiation induction, both CM-MSCs and DC-MSCs lost their proliferative ability even without the committed lineage. On the other side, once AM-MSCs and UC-MSCs commit to osteoblasts, they will lose their proliferative ability.

### Fibronectin and laminin in MSCs differentiation

**Fig. 5:**
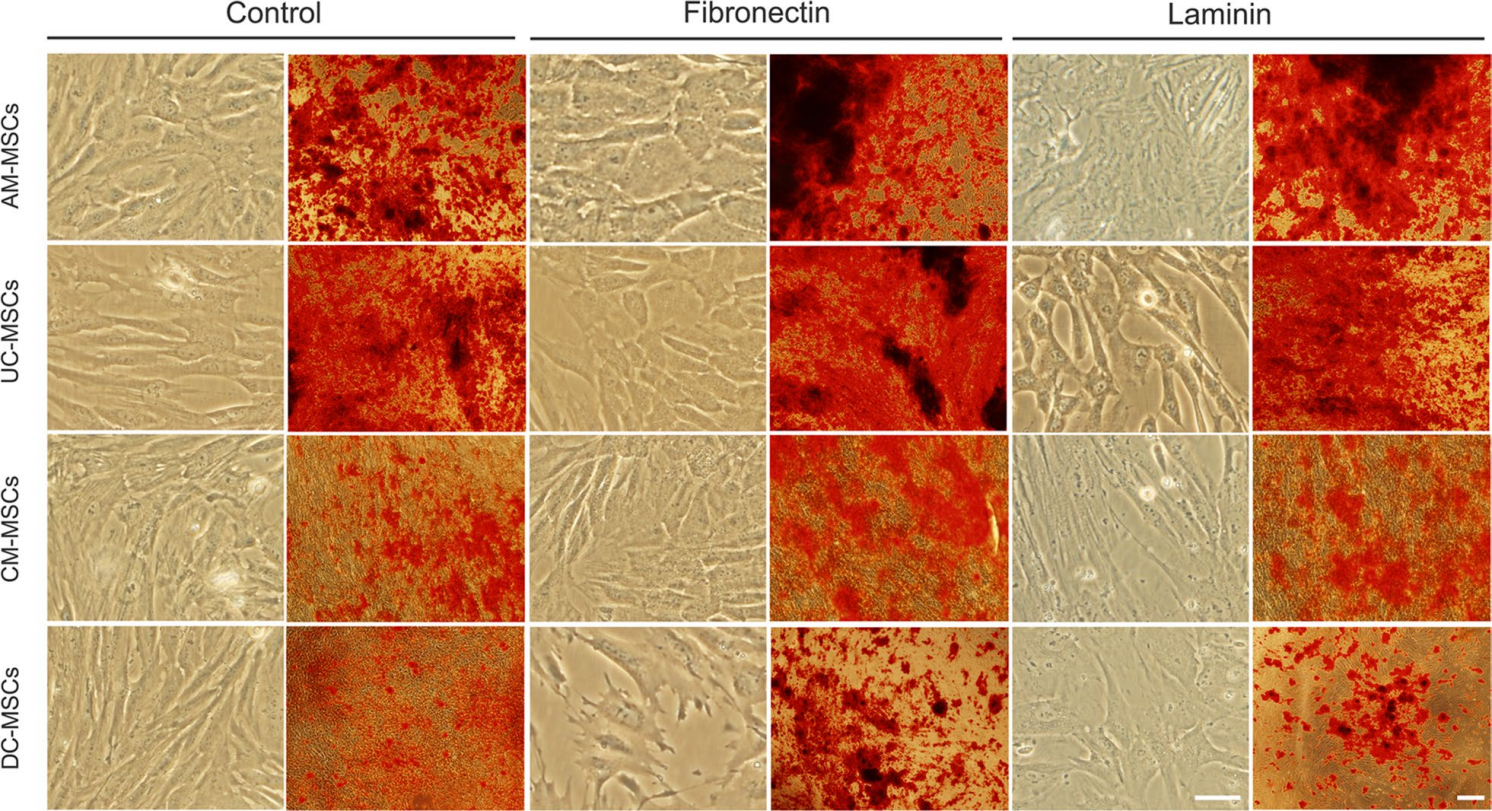
Fibronectin and laminin induced cell morphology changes and promoted osteogenic differentiation in AM-MSCs, UC-MSCs, CM-MSCs, and DC-MSCs. Morphologic analyses of the PD-MSCs have been cultured in tissue culture plastic, fibronectin or laminin with α-MEM. Alizarin red staining for analysis of the amount of calcium deposition in AM-MSCs, UC-MSCs, CM-MSCs, and DC-MSCs after 21 days of osteogenic differentiation on tissue culture plastic, fibronectin, or laminin. (Alizarin red staining pictures, Scale bar = 500 μm, magnification = ×40) (Morphology pictures, Scale bar = 200 μm, magnification = ×250)

**Fig. 6:**
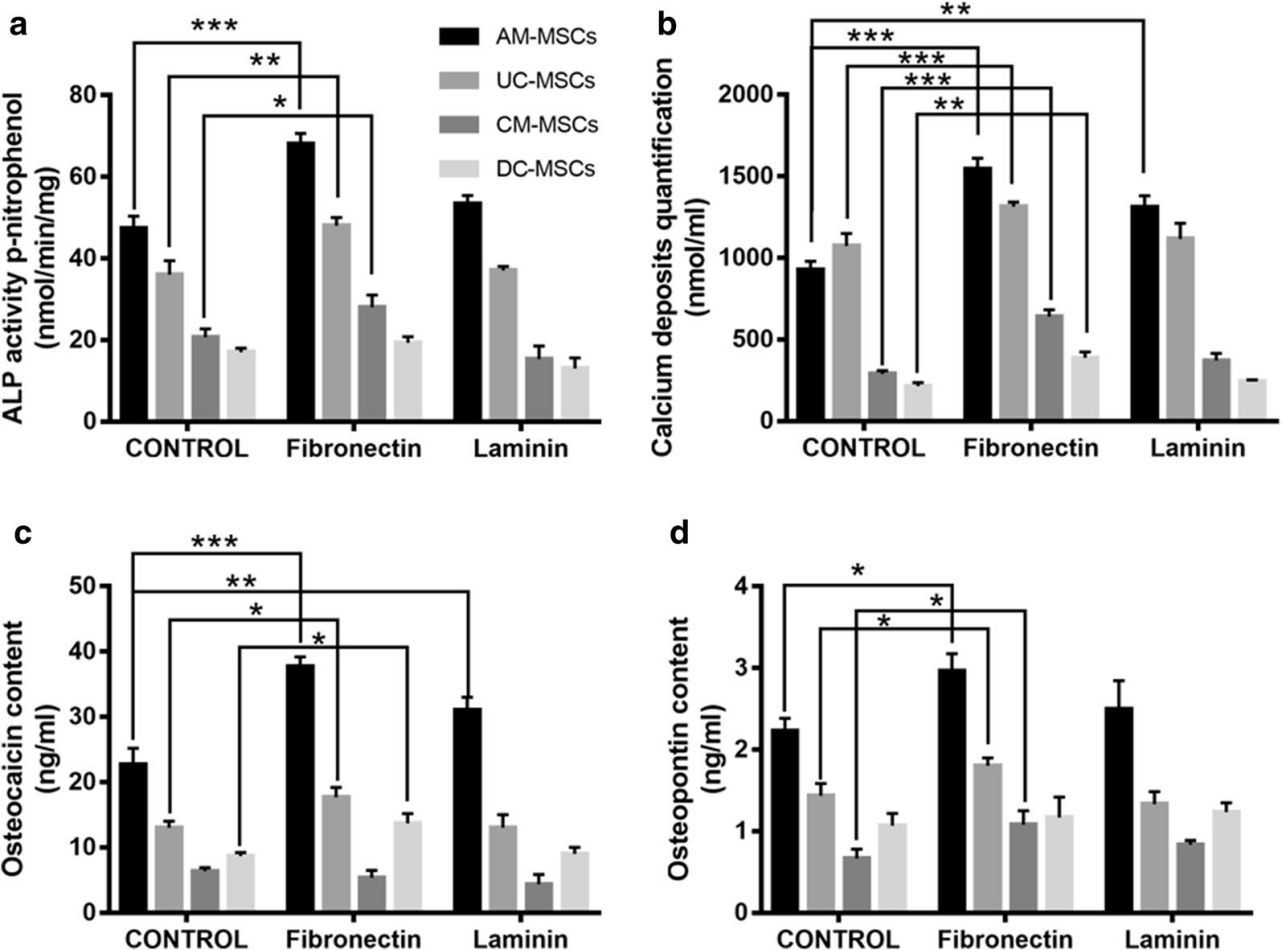
Evaluation of the surface modifications with fibronectin and laminin on the osteogenic commitment and differentiation in AM-MSCs, UC-MSCs, CM-MSCs, and DC-MSCs. **a** ALP activity and **b** calcium deposit levels were measured spectrophotometrically at days 21. **c** Osteocalcin and **d** osteopontin concentration were determined using an ELISA assay. n = 3/group, error bars represent the SD, *p < 0.05, **P ≤ 0.01; ***p ≤ 0.001 in the different groups compared with the control group using a One-way ANOVA test

Stem cells proliferation and differentiation are influenced by ECM components, which have been widely applied in tissue engineering. To determine the roles of ECM components on MSCs osteogenic differentiation, calcium deposits levels, ALP activity, the osteopontin and osteocalcin concentrations were determined. Notably, fibronectin and laminin provided dynamic microenvironments to regulate MSCs morphology, as shown in (Fig. 5), MSCs cultured on fibronectin-coated and laminin-coated plate for 24 h led to changes in cellular morphology. After 21 days of osteogenic differentiation, all of the stem cell types cultured on fibronectin stained positive for calcium deposition. The positive staining was greater than in the control groups. In contrast, the cells cultured on laminin displayed weak staining for calcium deposition when compared with the MSCs grown on the fibronectin, but no significant difference compared with the control. The AM-MSCs, UC-MSCs, and CM-MSCs cultured on fibronectin in an osteogenic medium experienced a dramatic increase in ALP activity, which is an early marker of osteoblast differentiation, compared with the control group (Fig. 6a). However, the laminin coated group displayed no difference compared to the control. Similarly, there was a marked increase in the calcium deposits levels in the AM-MSCs, UC-MSCs, CM-MSCs, and DC-MSCs cultured on fibronectin when induced by the osteogenic medium (Fig. 6b). In the laminin coated group, only the AM-MSCs exhibited a significant increase. As expected, the osteocalcin concentration expressed by the AM-MSCs, UC-MSCs, and DC-MSCs grew on fibrinogen was statistically greater than that of the control group. Only the AM-MSCs in the laminin coated group showed a dramatical increase (Fig. 6c). The AM-MSCs, UC-MSCs, and CM-MSCs cultured on fibronectin experienced an increasing trend in osteopontin concentration which is a late stage marker of osteoblast differentiation. There was no difference between the laminin coated group and the control group (Fig. 6d). Taken together, these data reveal that fibronectin not only promotes higher calcium deposit levels and ALP activity in AM-MSCs, UC-MSCs, and CM-MSCs, but also it accelerates the development of a mature osteoblast phenotype by enhancing the expression of osteopontin and osteocalcin. On the contrary, the results for the laminin coated group was not as significant as fibronectin group, and the laminin only had slight effects on the promotion of the expression of calcium deposit levels and osteocalcin concentration in the AM-MSCs.

### Fibronectin enhanced osteogenic differentiation via the Akt and ERK pathways

**Fig. 7:**
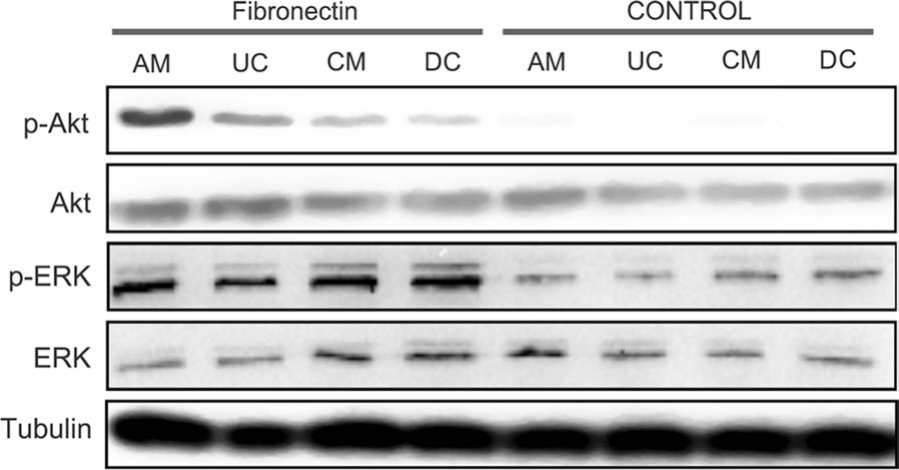
Fibronectin induced activation of Akt and ERK in AM-MSCs, UC-MSCs, CM-MSCs, and DC-MSCs to promote osteogenic differentiation. Western blot analysis of p-Akt, Akt, p-ERK, and ERK with and without fibronectin modification in the PD-MSCs. Tubulin was used as the internal control

Next, how fibronectin facilitated osteogenic differentiation in the AM-MSCs, UC-MSCs, CM-MSCs, and DC-MSCs was investigated. ECM, like fibronectin, effectively regulate cell adhesion and differentiation by activating signaling molecules such as Akt and ERK [24]. Therefore, to examine the involvement of Akt and ERK phosphorylation activation in the enhancement of osteogenic differentiation by fibronectin, a western blot assay was performed. As shown in (Fig. 7), cells treated with fibronectin led to a significant increase in the phosphorylation of Akt and ERK compared to the control. More specifically, the phosphorylation extent of Akt in the AM-MSCs and UC-MSCs was greater than in the CM-MSCs and DC-MSCs, which is consistent with the finding of the calcium deposit levels, ALP activity, and the osteopontin and osteocalcin concentration assays. Together, these findings suggest that fibronectin could promote osteogenic differentiation via enhanced phosphorylation of Akt and ERK in the AM-MSCs and UC-MSCs.

## Discussion

In the early stages of this type of research, efforts had focused on ways to use osteogenic cells, osteoinductive stimulators and biomaterial to develop viable substitutes for bone replacement. Although important advances have been achieved with stem cells and osteoinductive ECM, their applications have not been established largely because of limitations in the choice of cell sources that can efficiently differentiate into osteoblasts and the unpredictability of the complex compounds of the ECM proteins. Therefore, further studies are required to evaluate the differentiation potential of various stem cell sources and the osteoinductive ability of the ECM components.

Recently, several studies regarding osteoblast differentiation from different stem cells have been reported. In particular, Chen et al. showed that AM-MSCs induced in an osteogenic medium had significantly enhanced ALP expression and calcium deposition [25]. Also, it has been shown that UC-MSCs displayed osteogenic differentiation ability on polyglycolic acid (PGA) [26]. CM-MSCs and DC-MSCs also showed osteogenic differentiation potentials [27]. However, no study has evaluated differences in the osteogenic differentiation potentials of AM-MSCs, UC-MSCs, CM-MSCs and DC-MSCs cells. In this study, it was shown that MSCs isolated from different layers of the human placenta had various potentials to differentiate into osteogenic cells. The results of this study showed that AM-MSCs and UC-MSCs had a higher capacity to differentiate into osteoblasts as compared to CM-MSCs and DC-MSCs.

In addition, MSCs differentiation into the osteoblastic phenotype involves several stages, including expression of osteoprogenitor-specific markers, collagen synthesis, and ECM mineralization [28]. Therefore, the current study used not only an alizarin red staining assay, but also a qPCR assay to investigate stage-specific markers produced at different time points in the AM-MSCs, UC-MSCs, CM-MSCs, and DC-MSCs. The qPCR study showed that the AM-MSCs and UC-MSCs displayed a greater osteoprogenitor phenotype than the CM-MSCs and DC-MSCs at day 7, although subcultures of the CM-MSCs and DC-MSCs formed sufficient BMP-6 and Runx2 expression under osteogenic differentiation induction at day 14. Similarly, the AM-MSCs and UC-MSCs exhibited a better osteoblast and osteocyte phenotype than the CM-MSCs and DC-MSCs at days 14 and 21, indicating that the osteogenic potential and extent can be markedly different among cells. Currently, the cellular proliferation rate during the osteogenic differentiation process remains somewhat controversial [29, 30]. In this study, cell proliferation during osteogenic differentiation was assessed using an EdU labeling assay. However, the later stage of osteogenic differentiation induced matrix synthesis and mineralization influent of the EdU and Hoechst 33342 label efficiency. Therefore, the proliferation rate results were only obtained between days 0 and 7. The results revealed that once AM-MSCs and UC-MSCs commit to osteoblasts, they will lose their proliferative ability. However, CM-MSCs and DC-MSCs also lost their proliferative ability even without the committed to osteoprogenitor statue. Pluripotency genes, including OCT4 and SOX2, have shown to be expressed in MSCs and are downregulated upon differentiation, which promoting cell proliferation [31]. However, it remains to be clarified, whether downregulation of pluripotency genes after the osteogenic differentiation induction affect the proliferative ability of CM-MSCs and DC-MSCs.

Laminin [32] and fibronectin [33], instead of ECM complex compounds, are the most used compounds to enhance the osteogenic differentiation of stem cell in tissue engineering. In the present study, to evaluate the differentiation level among the AM-MSCs, UC-MSCs, CM-MSCs, and DC-MSCs, osteogenic differentiation markers, including calcium deposits levels, ALP activity, and the osteopontin and osteocalcin concentrations were analyzed. The results indicated that fibronectin, instead of laminin, enhanced the expression of osteopontin and osteocalcin only in AM-MSCs, and UC-MSCs, and it produced a higher ALP activity and calcium deposit levels in all of the cells types. This was consistent with other published findings that fibronectin promotes osteogenic differentiation and cell adhesion than any other ECM protein [34, 35]. Fibronectin seems to be a potential osteoinductive component, however, the molecular signaling pathways that fibronectin mediates to enhance osteogenic differentiation remain to be identified. The current study demonstrates that treatment of AM-MSCs, UC-MSCs, CM-MSCs, and DC-MSCs with fibronectin enhanced the phosphorylation of Akt and ERK. These findings suggest that Akt and ERK activation was associated with fibronectin-induced enhancement of osteogenic differentiation.

## Conclusions

**Fig. 8:**
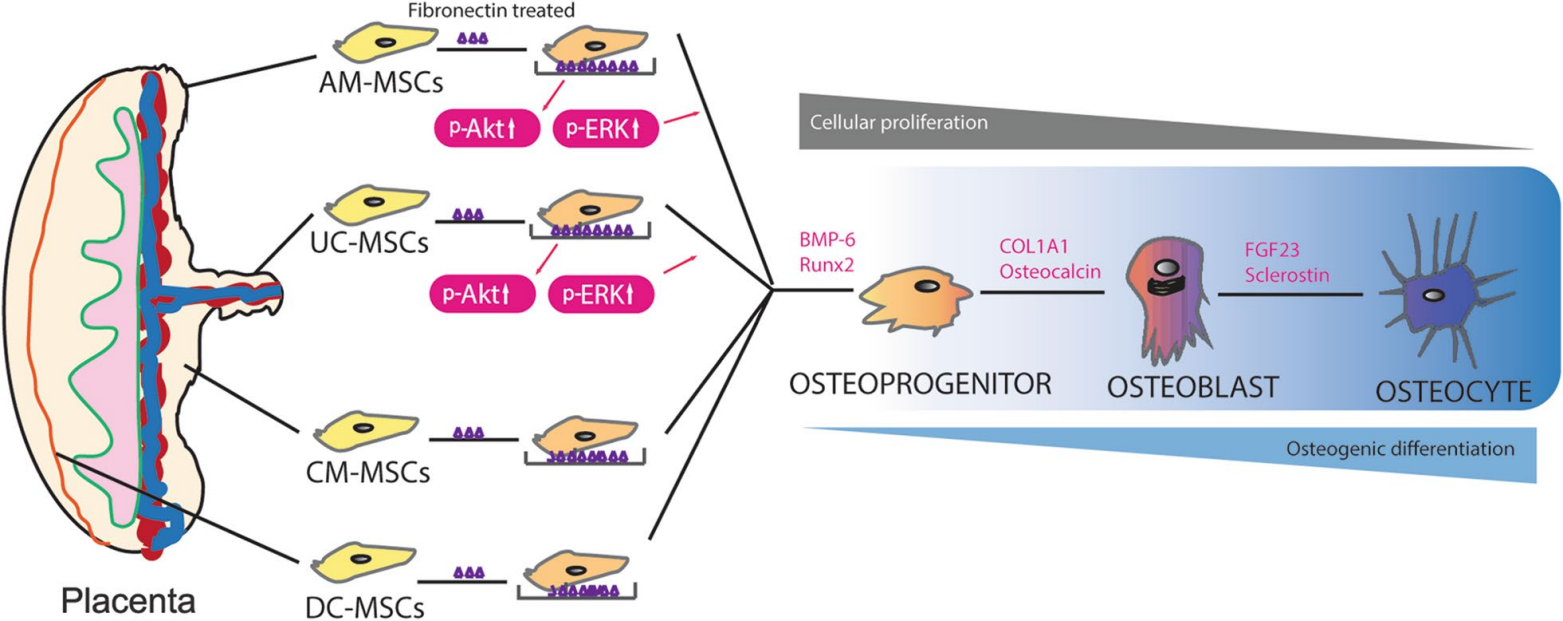
Osteogenic differentiation of the MSCs from the amniotic membrane (AM), umbilical cord (UC), chorionic membrane (CM), and decidua (DC). The MSCs cellular proliferation actives decreased significantly during differentiation into the early phase of the osteoblasts. The osteoprogenitor was derived from AM-MSCs, UC-MSCs, CM-MSCs, and DC-MSCs. These osteoprogenitor cells began to produce BMP-6 and Runx2. After that osteoprogenitor cells partially differentiated to osteoblasts, which are characterized by their markers COL1A1 and osteocalcin. At the end of the Osteogenic differentiation stage, osteoblast fully differentiated into osteocyte enhanced expressions of FGF23 and sclerostin

In summary, the results of this study indicate that AM-MSCs and UC-MSCs possess a higher osteogenic potential, which would make them the optimal stem cell source for bone tissue engineering. The results also, validated that fibronectin can enhance osteogenic differentiation in all four types of MSCs via the Akt and ERK pathways, which provides possible opportunities to modulate the osteogenic differentiation of MSCs to facilitate them clinical applications (Fig. 8).

## Methods

### Cell culture

Human AM-MSCs, UC-MSCs, CM-MSCs, and DC-MSCs were provided by Sichuan mesenchymal stem cells bank. Cells cultured in α-MEM (Invitrogen, Carlsbad, CA, USA) supplemented with 10% heat-inactivated FBS (Gibco, Carlsbad, CA, USA), 2 mM l-glutamine (Gibco), 100 U/ml penicillin, and 100 μg/ml streptomycin (Gibco), in a 37 °C, 5% CO_2_ incubator (Sanyo, Osaka, Japan). Cell growth medium was changed every 3 days. Cells were passaged with 0.125% trypsin (Gibco) at 75% confluence.

### Osteogenic differentiation

To induce osteogenic differentiation of AM-MSCs, UC-MSCs, CM-MSCs, and DC-MSCs, cells were cultured in osteogenic differentiation media with StemPro Differentiation Kit (Gibco) according to the manufacturer’s instructions. AM-MSCs, UC-MSCs, CM-MSCs, and DC-MSCs were seeded in 6-well plates or 96-well plates, osteogenic differentiation media were changed every 3 days. Alizarin Red (Sigma-Aldrich, St. Louis, MO, USA) staining was used to detect calcium deposits.

### ALP activity measurement

The ALP activity was measured by quantitative alkaline phosphatase ES characterization kit (Millipore) according to the manufacturer’s protocols. Under alkaline conditions (pH > 10), ALP can catalyze the hydrolysis of *p*-nitrophenyl phosphate (*p*-NPP) into phosphate and *p*-nitrophenol. The amount of *p*-nitrophenol released is related to the amount of alkaline phosphatase in the reaction. Add *p*-NPP substrate solution and read the absorbance at 405 nm on a spectrophotometer (Bio-rad, Laboratories, Hercules, CA, USA).

### Calcium depos-its quantification

Osteogenesis quantitation kit (Millipore) was used for quantification of calcium depos-its. After alizarin red staining, 10% acetic acid was added to collect the cells and incubated at 85 °C for 10 min. Centrifuge the cell mixture at 16,000*g* for 20 min. Then, remove the supernatant and adjust pH value to 4.1–4.5 with 10% ammonium hydroxide. Read the absorbance at 405 nm on a spectrophotometer (Bio-rad).

### Elisa assay

To analyze the accumulative release of osteopontin and osteocalcin, cell culture supernatant was harvested for analyses using ELISA assay kit (abcam). Briefly, 200 μl of cell culture supernatant was added to 96-well plates that were coated with a monoclonal antibody specific to osteopontin or osteocalcin, incubated for 3 h. After washing with PBS, the antibody was added to each well, the plates were incubated for 1 h, washed with wash buffer, and substrate solution was added. Then, the concentration of cytokine was calculated by reading the absorbance at 450 nm on a spectrophotometer (Bio-rad).

### RNA extraction and qRT-PCR

During osteogenic differentiation of AM-MSCs, UC-MSCs, CM-MSCs, and DC-MSCs at days 0, 7, 14 and 21, total cellular RNA was extracted by using RNeasy mini kit (Qiagen, Venlo, Netherlands). To remove genomic DNA contamination, DNase I (Invitrogen) digestion was performed. cDNA was synthesized from total cellular RNA using SuperScript III first-strand synthesis system (Invitrogen). Quantitative reverse transcription-polymerase chain reactions (qRT-PCR) reactions were performed using SYBR green master mix (ABI, Invitrogen) and 7300 real-time PCR system (ABI). The mRNA expression levels were normalized using β-actin RNA as internal control. The sequences of primers are shown in Additional file 1: Table S1.

### EdU labeling assay

Cells cultured on poly-lysine-coated coverslips in a 24-well plates and incubated at 37 °C for 8 h. 10 μM EdU solution (Invitrogen) was added to the cell culture medium treated for 6 h. Then coverslips were fixed using PBS with 3.7% formaldehyde and permeabilization by a 0.5% Triton X-100 solution. 0.5 ml of Click-it plus reaction cocktail (Invitrogen) was added to each coverslip and incubated for 30 min. Hoechst 33342 (Invitrogen) was applied to show nucleus. Coverslips were preserved with mounting media and imaged by fluorescence microscopy (Leica).

### Western blotting

After 21 days osteogenic differentiation of AM-MSCs, UC-MSCs, CM-MSCs, and DC-MSCs, the total cellular protein was extracted using the cell lysis buffer (Beyotime), and concentrations were determined by Bradford protein assay kit (Bio-Rad). Proteins were loaded in SDS-AGE gel and electrophoresed at 80 V for 30 min and 140 V for 60 min. Then, proteins were transferred from gel to nitrocellulose membrane using a trans-blot electrophoretic transfer kit (Bio-Rad). Membranes were blocked in 5% skim milk in TBST buffer for 60 min and incubated with primary antibodies osterix (1:3000, ab94744, abcam 45kd), collagen I (1:3000, ab34710, abcam 125kd), osteopontin (1:2000, ab166709, abcam 65kd), osteocalcin (1:2000, ab93876, abcam 11kd), Tubulin(1:4000, ab4074, abcam 50kd), phosphate-Akt (1:2000, 193H12, Cell signaling technology), Total-Akt (1:2000, C67E7, Cell Signaling Technology), phosphate-Erk1/2 (Thr202/Tyr204, Cell Signaling Technology), total- ERK1/2 (1:2000, Cell Signaling Technology). After washing with TBST buffer, the membranes were incubated with HRP goat anti-mouse IgG (1:3000, Beyotime) or HRP goat anti-rabbit IgG (1:3000, Beyotime). Membranes were then incubated with pierce ECL western blotting substrate (Thermo fisher) and then imaged using chemidoc imaging system (Bio-Rad).

### Statistical analysis

We used the GraphPad Prism software (v7) to conduct statistical analysis (GraphPad Software). Data were expressed as the mean ± SD. Unless otherwise noticed, differences between two experimental groups were applied using an unpaired two-tailed Student’s t-test. For comparison of more than three groups, one-way ANOVA was applied. Results were considered statistically significant with p values: ***p < 0.001**p < 0.01; *p < 0.05.

## Additional file

**Additional file 1: Table S1.** The sequences of qPCR primers.

## Abbreviations

AM: amniotic membrane
UC: umbilical cord
CM: chorionic membrane
DC: decidua
MSCs: mesenchymal stem cells
ALP: alkaline phosphatase
ADSCs: adipose tissue-derived mscs
BM-MSCs: bone marrow mscs
ECM: extracellular matrix
