# Supplementary material for: Comparison of osteogenic differentiation capacity in mesenchymal stem cells derived from human amniotic membrane (AM), umbilical cord (UC), chorionic membrane (CM), and decidua (DC)
Source: Cell Biosci. 2019 Feb 11;9:17. doi: 10.1186/s13578-019-0281-3 (PMC6371545; doi:10.1186/s13578-019-0281-3)
Supplement: Supplementary file 1 — Additional file 1: Table S1. The sequences of qPCR primers. [file 13578_2019_281_MOESM1_ESM.docx]

**The qPCR primers**

| Name | Forward 5’-3’ | Reverse5’-3’ |
| --- | --- | --- |
| BMP-6 | GCTCAACCGCAAGAGCCTTC | TGTCGTACTCCACCAGGTTC |
| Runx2 | CAGATGATGACACTGCCACCT | GCATTCGTGGGTTGGAGAAG |
| COL1A1 | AGTGGTTTGGATGGTGCCAA | GCACCATCATTTCCACGAGC |
| Osteocalcin | GTGCAGCCTTTGTGTCCAAG | TCAGCCAACTCGTCACAGTC |
| FGF23 | GAGCCTATCCCAATGCCTCC | CTTGTGGATCTGCAGGTGGT |
| Sclerostin | CAGCCTTCCGTGTAGTGGAG | GCTGTACTCGGACACGTCTT |

Table S1. The sequences of qPCR primers.
